# The effect of micronutrient levels on leukocyte telomere length: A Mendelian randomization study

**DOI:** 10.1097/MD.0000000000049408

**Published:** 2026-07-03

**Authors:** Hanyu Zhu, Manman Chen, Yaowen Wang, Xiuqiang Zhang, Chunlin Li, Hehe Wang

**Affiliations:** a Department of Otolaryngology Head and Neck Surgery, First Affiliated Hospital of Ningbo University, Ningbo, Zhejiang Province, China.

**Keywords:** copper, leukocyte telomere length (LTL), Mendelian randomization (MR), vitamin B6

## Abstract

Leukocyte telomeres play a crucial role in maintaining genomic stability and human health. This study used Mendelian randomization (MR) to investigate the effects of vitamin A, vitamin B6, vitamin B12, vitamin C, vitamin D, vitamin E, calcium, carotenoids, copper, folate, iron, magnesium, potassium, selenium, and zinc on leukocyte telomere length (LTL). Summary statistics were obtained from genome-wide association studies for both exposure and outcome data. This study performed MR analysis on each exposure–outcome pair. The weighted median, MR-Egger, and MR-PRESSO methods were used for performing sensitivity analyses. The MR-Egger regression intercept was employed for assessing directional pleiotropy, and the MR-PRESSO test for analyzing individual outliers due to pleiotropic effects. The inverse-variance weighted method is our primary estimation tool. Univariable MR analyses identified potential associations between genetically predicted copper and vitamin B6 levels and LTL. However, multivariable Mendelian randomization analysis revealed that only copper retained a significant positive causal effect on LTL (*P* < .05), whereas the association with vitamin B6 was attenuated and became non-significant after adjustment. This study provides genetic evidence consistent with an association between higher copper levels and longer LTL. While vitamin B6 showed a nominal association in univariable analysis, it was not an independent predictor in the multivariable model. These findings highlight a potential role of copper in telomere biology, although further mechanistic studies are warranted to validate these results.

## 1. Introduction

Telomeres are repetitive DNA sequences (TTAGGG) located at the ends of chromosomes, safeguarding the integrity of the information-carrying DNA and thus playing a crucial role in maintaining genomic stability.^[1,2]^ Although telomeres prevent the loss of base pairs in chromosomal DNA during successive cell divisions, their length reduces with each normal mitotic DNA replication.^[3]^ Cells will lose their ability to divide further and enter a senescent state once telomeres are shortened to a critical length. Consequently, telomere length has emerged as an important biomarker of biological aging.^[4,5]^ As demonstrated, there is a negative correlation between telomere length and age. As telomere length decreases with age, it was indirectly suggested that telomere length serves as a cellular marker of biological aging.^[6]^ A previous study has shown that telomeres remain stable from childhood to adolescence but gradually shorten thereafter, despite significant variability among individuals in both initial telomere length and the rate of its subsequent shortening.^[7]^ Njajou et al^[8]^ believe that leukocyte telomeres play a crucial role in aging research. They found that leukocyte telomere length (LTL) shows a significant positive correlation with healthy years. It is indicated that LTL is a potential biomarker of healthy aging.^[8]^ However, factors such as chronic inflammation,^[9]^ oxidative stress,^[10]^ smoking, and obesity^[11]^ can accelerate the shortening of LTL. Numerous studies have shown that shortened LTL is associated with an increased risk of age-related diseases, such as hypertension, diabetes, ischemic heart disease, and mortality related to these conditions.^[12]^ Previous research by Rode et al^[13]^ also indicated a significant positive correlation between shorter LTL and mortality. Thus, there is a clear relationship between LTL and human health and aging. As essential components of the human body, the relationship between micronutrients and LTL is a significant focus of recent research.

In this study, to comprehensively explore the intersection of nutrition and genomic stability, we selected 15 essential micronutrients based on a combination of their established biological roles in oxidative stress, DNA maintenance, and aging, as well as the availability of high-quality genome-wide association studies (GWAS) data. This selection allowed for both hypothesis-driven investigation of nutrients with prior evidence linking them to telomere biology (e.g., copper, B vitamins, and antioxidants) and an exploratory scan of other micronutrients with less well-characterized relationships.

These included vitamin A, vitamin B6, vitamin B12, vitamin C, vitamin D, vitamin E, calcium, carotene, copper, folate, iron, magnesium, potassium, selenium, and zinc. These micronutrients can be obtained from food and water sources.^[14]^ It is crucial for ensuring adequate levels of micronutrients in the body, while excessive intake may cause risks.^[15]^ The involvement of these micronutrients in various human health conditions makes them crucial in disease states. Previous study has shown that potassium may lower ischemic heart disease risk by affecting BDH2 and C1R plasma proteins. Vitamin B12 may elevate coronary atherosclerosis and cardiovascular death risks by decreasing VPS29 and PSME1 protein levels, while vitamin C may decrease cardiac arrest risk by inhibiting TPST2 protein expression. In addition, potassium reduces ischemic heart disease risk by decreasing 4-methoxyphenyl sulfate levels.^[16]^ Fekete et al^[17]^ have found that minerals such as zinc, copper, iron, magnesium, manganese, selenium, and calcium may exert beneficial effects on chronic obstructive pulmonary disease through research on chronic obstructive pulmonary disease and micronutrients. Maintaining optimal levels of these micronutrients may aid in modulating inflammatory processes, reducing oxidative stress, and improving lung function. Ahoon et al^[18]^ have discovered that combining vitamin D with standard asthma treatment can effectively improve clinical symptoms and enhance the quality of life of asthma patients. Collet et al^[19]^ found that levels of K, Rb, Se, Fe, P, Si, S, δ65Cu, Cu, the S/Se ratio, and the Cu/Zn ratio were independently associated with the survival rates of sarcoma patients by correlating the concentrations of metals and micronutrients in the serum of sarcoma patients with survival data.

While prior observational studies have explored the association between specific micronutrients and telomere length – for example, studies suggesting a link between dietary copper intake and LTL^[20,21]^ – results have been susceptible to inherent limitations. Observational data on food and nutrients are frequently derived from the Food Frequency Questionnaire,^[22]^ which is subjective and prone to measurement error. Furthermore, traditional epidemiological studies are often hindered by residual confounding and reverse causality, making it difficult to establish true causal relationships.

To address these limitations, our study adopts Mendelian randomization (MR), an objective statistical approach based on Mendel’s Second Law. It uses genetic variants to assess the causal relationship between exposure and outcome. As alleles follow the principle of random distribution and the law of independent assortment during gamete formation, MR can reduce confounding factors often encountered in traditional epidemiological studies and reverse causality, thereby minimizing research bias.^[23,24]^ Therefore, we can use MR methods based on GWAS to further explore the association between micronutrients and LTL.

## 2. Materials and methods

### 2.1. Study design

**Figure 1.**
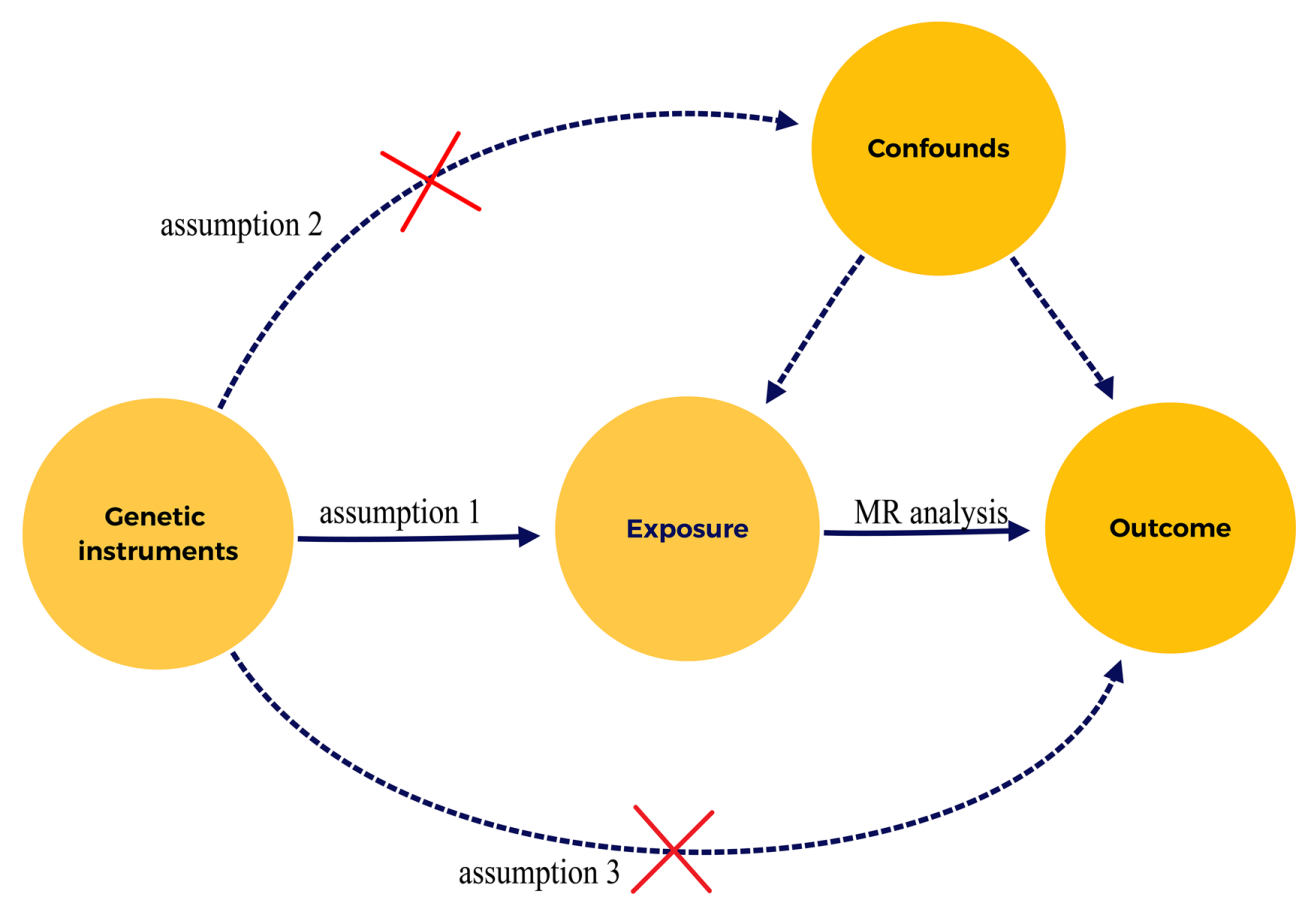
The conceptual MR framework. MR = Mendelian randomization.

The study makes use of MR and 2-sample MR.^[25]^ It was designed rigorously based on 3 crucial assumptions: The genetic variant is directly and strongly associated with exposure (micronutrient levels); the genetic variant is unrelated to potential confounding factors; and the genetic variant affects the outcome only through exposure. The detailed MR framework is shown in Figure 1.

### 2.2. Micronutrient GWAS data source

We employed summary statistics from extensive GWAS for both exposure and outcome data. Detailed information regarding the GWAS datasets is provided in Table 1. The exposure data included 15 micronutrients. To mitigate population stratification bias, we utilized summary statistics primarily from European ancestry populations. The outcome dataset for LTL was obtained from a large-scale GWAS involving 472,174 participants (ieu-b-4879). We assessed potential sample overlap between exposure and outcome datasets. Where overlap was unavoidable (e.g., both involving UK Biobank participants), we relied on strong *F*-statistics to minimize weak instrument bias, although we acknowledge this as a limitation. Our study used only aggregated, publicly available data, so ethical approval was not needed.^[26]^

**Table 1:** Detailed information regarding the GWAS datasets.

Trait	ID	N	Number of SNPs	Population	Author	Year
Copper	ieu-a-1073	2603	2543646	European	Evans	2013
Calcium	ukb-b-8951	64979	9851867	European	Ben Elsworth	2018
Carotene	ukb-b-16202	64979	9851867	European	Ben Elsworth	2018
Folate	ukb-b-11349	64979	9851867	European	Ben Elsworth	2018
Iron	ukb-b-20447	64979	9851867	European	Ben Elsworth	2018
Magnesium	ukb-b-7372	64979	9851867	European	Ben Elsworth	2018
Potassium	ukb-b-17881	64979	9851867	European	Ben Elsworth	2018
Selenium	ieu-a-1077	2603	2543617	European	Evans	2013
Vitamin A	ukb-b-9596	460351	9851867	European	Ben Elsworth	2018
Vitamin B12	ukb-b-19524	64979	9851867	European	Ben Elsworth	2018
Vitamin B6	ukb-b-7864	64979	9851867	European	Ben Elsworth	2018
Vitamin C	ukb-b-19390	64979	9851867	European	Ben Elsworth	2018
Vitamin D	ukb-b-18593	64979	9851867	European	Ben Elsworth	2018
Vitamin E	ukb-b-6888	64979	9851867	European	Ben Elsworth	2018
Zinc	ieu-a-1079	2603	2543610	European	Evans	2013
Telomere length	ieu-b-4879	472174	20134421	European	Codd	2021

GWAS = genome-wide association studies, SNP = single-nucleotide polymorphism.

### 2.3. Selection of instrumental variables

To obtain a sufficient number of instrumental variables (IVs), single-nucleotide polymorphisms (SNPs) were selected using a significance threshold of *P* < 5 × 10^−6^, a standard approach in nutritional MR where genome-wide significant SNPs are often scarce. This threshold represents a trade-off between including enough instruments and the increased risk of pleiotropy; we therefore also assessed sensitivity using more lenient thresholds and examined pleiotropy through multiple methods. We clumped SNPs using a strict threshold (*r*^2^ < 0.001, kb = 10,000) based on the European 1000 Genomes reference panel. To ensure instrument strength, we calculated the variance explained (*R*^2^) and the *F*-statistic for each SNP. Only SNPs with an *F*-statistic >10 were retained to avoid weak instrument bias. Detailed SNP characteristics, including effect alleles, beta coefficients, standard error, and *P* values, are provided in Table S1, Supplemental Digital Content 1.^[27]^

### 2.4. Statistical analysis

Our study performed MR analysis on each exposure-outcome pair, using 15 micronutrients as exposures and telomere length phenotypes as outcomes. The study utilized the inverse-variance weighted (IVW) method, applying both fixed-effects and random-effects models.^[28]^ To account for multiple testing across the 15 micronutrient exposures, we applied the Benjamini-Hochberg false discovery rate correction. A false discovery rate-adjusted *P*-value (*q*-value) <.05 was considered significant, while a raw *P*-value <.05 with *q* >0.05 was considered suggestive evidence.^[29]^ For micronutrients showing significant or suggestive associations in univariable analysis (copper and vitamin B6), we performed Multivariable Mendelian Randomization (MVMR), including both exposures simultaneously to estimate their independent effects on LTL. Given that other micronutrients did not show evidence of association in univariable analyses, they were not included in the MVMR model to avoid overfitting and maintain statistical power. We assessed the strength of instruments in the MVMR setting using the Sanderson-Windmeijer conditional *F*-statistic to ensure that the genetic variants were strong predictors of the exposure conditional on the other covariates. The results of the *F*-statistic were shown in Table S2, Supplemental Digital Content 2. All statistical analysis were performed using R version 4.3.1.^[30,31]^ Estimates were converted to odds ratios (ORs) with 95% confidence intervals (CIs).

## 3. Results

After rigorous quality control for IVs, we identified 188 SNPs. All SNPs associated with these metabolites exhibited *F*-statistics greater than 10, indicating strong IV power. We then employed MR analysis to assess the potential causal relationship between these 188 SNPs and LTL.

**Figure 2.**
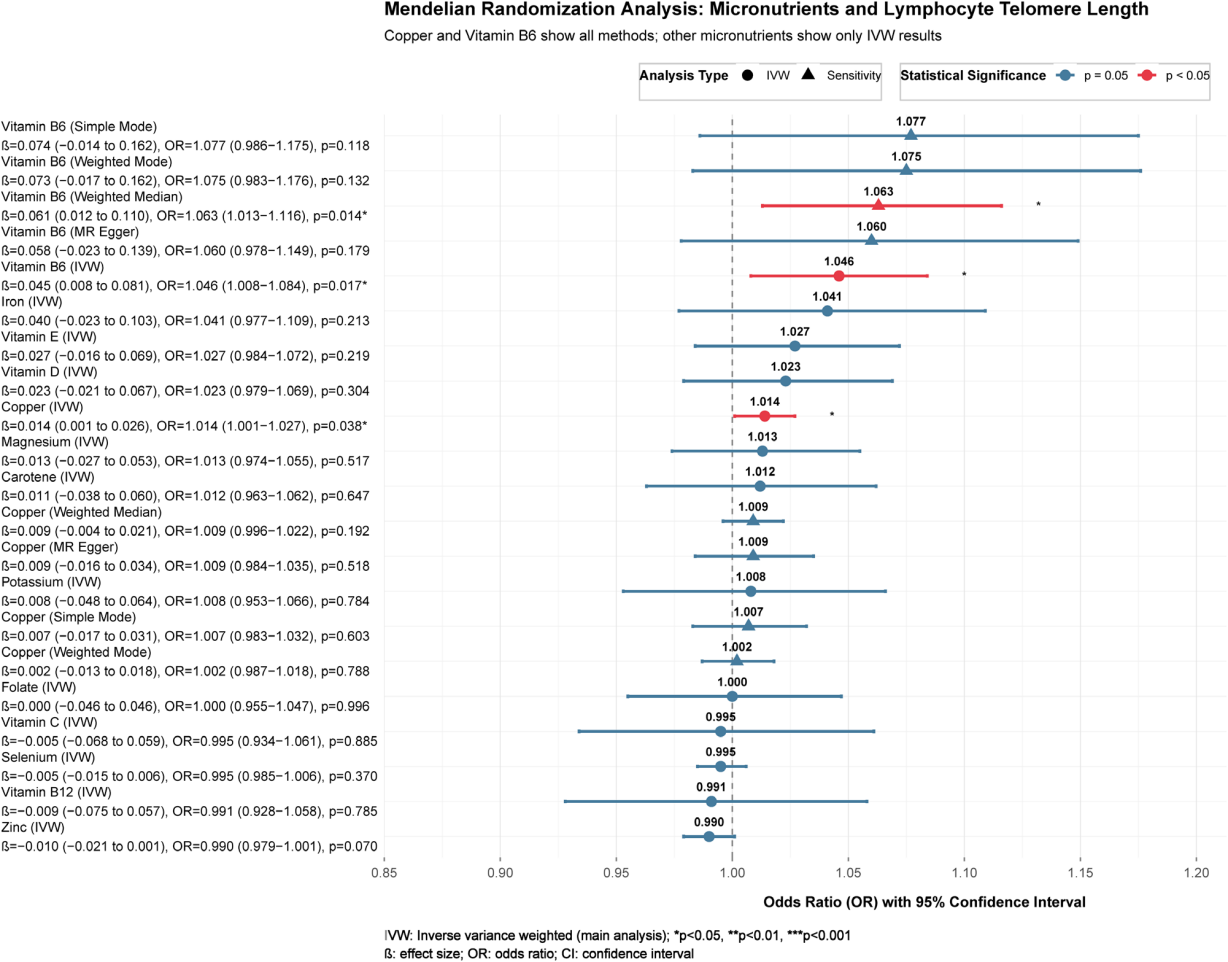
Forest plot of Mendelian randomization estimates for the association between 15 micronutrients and leukocyte telomere length.

The IVW method initially identified copper and vitamin B6 as potential causal factors affecting telomere length. As illustrated in the forest plot (Fig. 2), genetically predicted copper levels demonstrated a significant positive association with LTL using the IVW method (OR = 1.014, 95% CI: 1.001–1.027, *P* = .038; β = 0.014). This indicates that for every standard deviation increase in genetically predicted copper, LTL increases by 0.014 standard deviations. Vitamin B6 also showed a significant positive association in the primary IVW analysis (OR = 1.046, 95% CI: 1.008–1.084, *P* = .017; β = 0.045) and the Weighted Median method (OR = 1.063, 95% CI: 1.013–1.116, *P* = .014). However, consistent evidence of causal association was not observed for the other 13 micronutrients, such as iron (OR = 1.041, *P* = .213), zinc (OR = 0.990, *P* = .070), or magnesium (OR = 1.013, *P* = .517). In addition, the estimates from IVW, MR-Egger, and weighted median methods were consistent in both direction and magnitude, as shown in Figure 2.

The plot displays the effect estimates (beta [β] and OR) for all micronutrients using the IVW method. For the significant candidates, copper and Vitamin B6, sensitivity analyses (MR-Egger, Weighted Median, Weighted Mode, Simple Mode) are also shown. Error bars represent 95% CIs. *P < .05. Figure 2 also displays the sensitivity analyses for the significant, potential candidates. For copper, the direction of effect was consistent across MR-Egger (β = 0.009) and weighted median (β = 0.009) methods, although these did not reach statistical significance (*P* > .05).

We interpreted the non-significant MR-Egger intercept for copper with caution. Given the limited number of IVs (n = 6), the statistical power to detect horizontal pleiotropy is low. Therefore, while no pleiotropy was detected, we cannot rule out its presence entirely. For vitamin B6 (n = 17), the results were robust across the weighted method, though the MR-Egger estimate had wider CIs.

**Figure 3.**
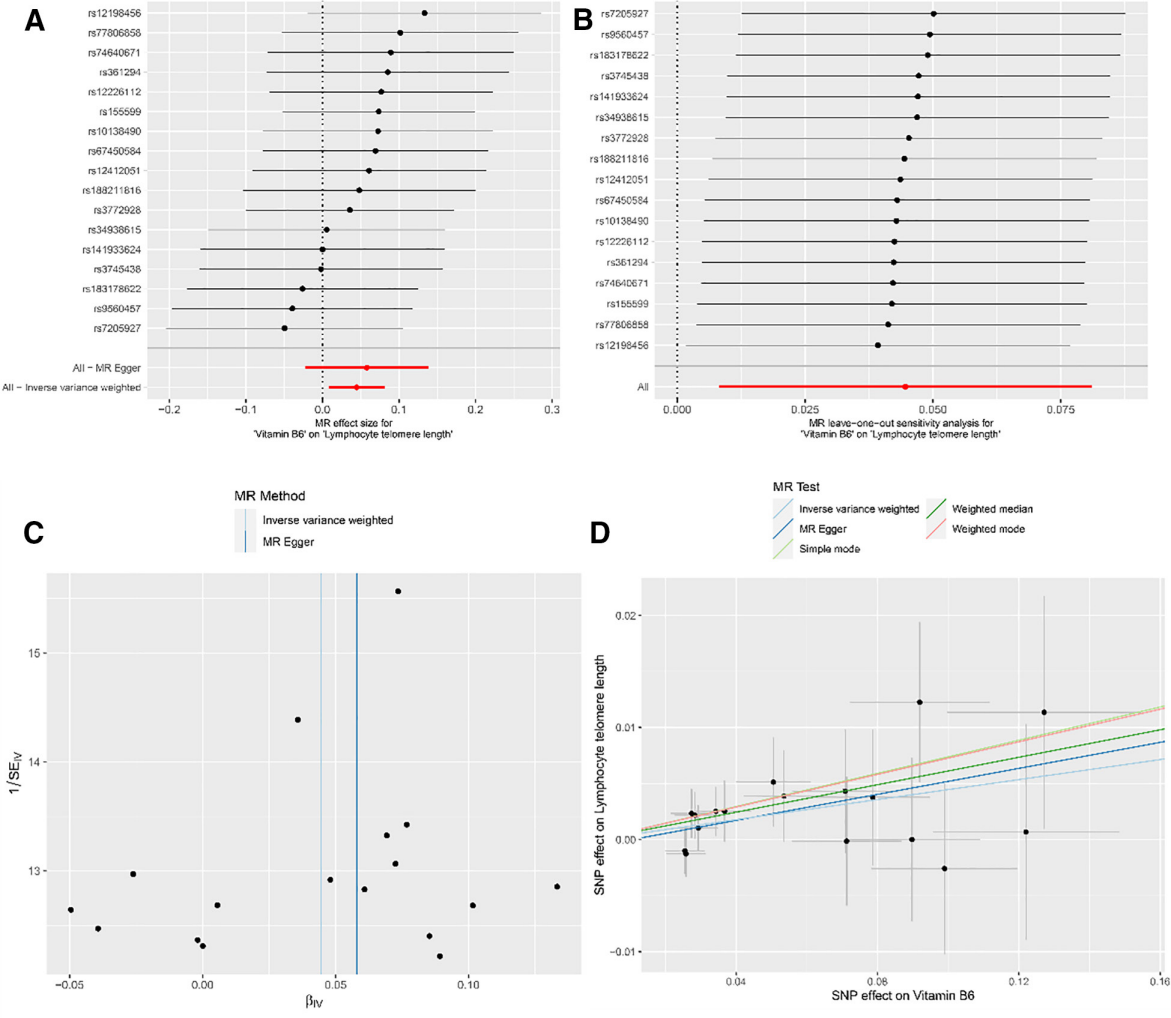
Four plots of vitamin B6 levels-related leukocyte telomere length: (A) forest plot; (B) leave-one-out plot of the overall IVW estimate; (C) funnel plots; (D) scatter plot. IVW = inverse-variance weighted, MR = Mendelian randomization, SNP = single-nucleotide polymorphism.

**Figure 4.**
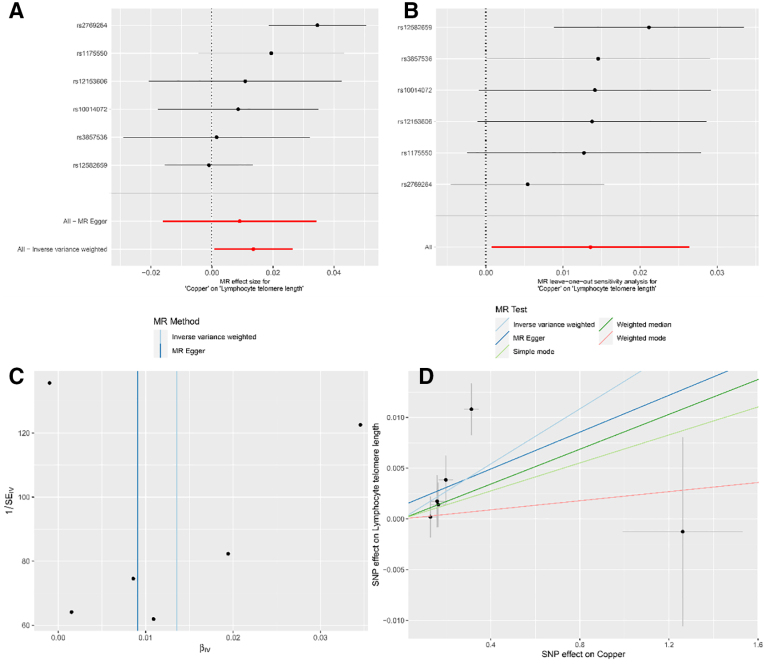
Four plots of copper levels-related leukocyte telomere length: (A) forest plot; (B) leave-one-out plot of the overall IVW estimate; (C) funnel plots; (D) scatter plot. IVW = inverse-variance weighted, MR = Mendelian randomization, SNP = single-nucleotide polymorphism.

Moreover, the results illustrated that there was no bias introduced in the estimation of MR due to a single SNP, as presented in Figures 3 and 4. Figure 2 presents the MR analysis results of copper and vitamin B6 as a forest plot.

**Figure 5.**
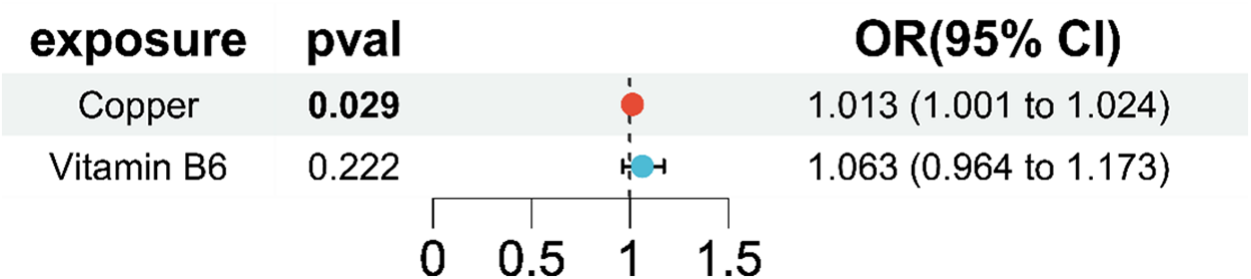
Copper is an independent influencing factor of leukocyte telomere length, but vitamin B6 is not. CI = confidence interval, OR = odds ratio.

These findings suggest that copper and vitamin B6 are promising candidates for further investigation of their potential causal effects on telomere length using MVMR analysis with the IVW method. The results revealed that copper can directly affect telomere length independently of other micronutrients (Fig. 5).

## 4. Discussion

This study employed large-scale GWAS summary statistics and MR analysis to comprehensively investigate the causal relationships between 15 micronutrients and LTL.^[32,33]^ To our knowledge, this is one of the first studies to apply MVMR to disentangle the independent effects of correlated micronutrients on telomere biology.^[34]^ Our primary finding indicates that genetically predicted higher copper levels are causally associated with longer LTL.^[35]^ While vitamin B6 initially showed a positive association in univariable analysis, this effect was attenuated and became non-significant after adjusting for copper in the multivariable model, suggesting it is likely not an independent causal factor.^[36]^

Our findings regarding copper provide genetic support for previous observational epidemiology. For instance, a cross-sectional study by Lin et al^[20]^ reported a positive association between dietary copper intake and telomere length in a large population sample. Similarly, Gong et al^[21]^ found consistent patterns in observational cohorts. However, traditional observational studies are often limited by residual confounding (e.g., socioeconomic status, overall healthy diet patterns) and measurement errors inherent in food frequency questionnaires.^[37]^ By utilizing MR, which exploits the random allocation of genetic variants at conception, our study minimizes these biases.^[38]^ The consistency between our genetic results and prior observational findings strengthens the evidence that physiological copper status plays a genuine role in telomere maintenance, rather than being a mere artifact of confounding.^[24]^

Although our MR analysis relies on genetic associations and cannot directly test biological mechanisms, several pathways linking copper to telomere maintenance are biologically plausible. The most prominent hypothesis concerns oxidative stress. Telomeres, rich in guanine, are highly susceptible to oxidative damage, which accelerates telomere attrition during cell division.^[39,40]^ Copper serves as an essential catalytic cofactor for Cu, Zn-superoxide dismutase (SOD1), a critical antioxidant enzyme located in the cytoplasm and mitochondrial intermembrane space.^[41]^ SOD1 catalyzes the dismutation of superoxide radicals into oxygen and hydrogen peroxide, thereby reducing the cellular oxidative burden.^[42]^ It is hypothesized that genetically supported optimal copper levels may enhance SOD1 activity, protecting telomeric DNA from oxidative insults and slowing the rate of shortening.^[43]^ This mechanistic link aligns with our finding of a protective effect of copper on LTL. The magnitude of the copper effect observed (0.014 SD increase in LTL per SD increase in genetically predicted copper) is modest. In the context of telomere biology, even small increments in LTL have been associated with reduced risk of age-related diseases in large epidemiological studies.^[12]^ However, whether such a modest change is biologically meaningful at the individual level remains uncertain and warrants further investigation. It is also important to note that LTL, as measured in GWAS, reflects cross-sectional variation rather than longitudinal attrition; therefore, our findings pertain to steady-state differences rather than rates of change over time.

Regarding vitamin B6, our univariable results initially aligned with the general understanding that B vitamins support genomic stability through the one-carbon metabolism pathway.^[44]^ However, the loss of significance in the MVMR analysis highlights the complexity of nutritional epidemiology. It is possible that the initial association was driven by pleiotropic genetic variants or high correlation with other traits.^[45]^ This underscores the necessity of using multivariable approaches to verify independent causal effects, preventing potential false-positive conclusions about specific nutrients.^[34,46]^

It is crucial to interpret these findings within the context of the MR methodology. Our results reflect the effects of lifelong, steady-state variation in serum copper levels determined by genotype, rather than the acute effects of dietary intake or supplementation in adulthood.^[47]^ Therefore, while our study supports a biological link between copper and telomere length, these findings should not be directly translated into clinical recommendations for copper supplementation without further validation from randomized controlled trials.^[48]^ Excessive copper intake can lead to toxicity and oxidative damage; thus, the “beneficial” effect observed here likely reflects physiological levels within a homeostatic range.^[49]^

This study has several strengths, including the use of large-scale GWAS data for LTL (N > 470,000)^[50]^ and the rigorous application of sensitivity analyses (MR-Egger, weighted median, MR-PRESSO) and MVMR.^[31,51]^ However, limitations must be acknowledged. First, the number of IVs for copper was relatively small (n = 6), which limits the statistical power of sensitivity analyses to detect subtle horizontal pleiotropy.^[52]^ Second, the study population was primarily of European ancestry, which minimizes population stratification bias but limits the generalizability of our findings to other ethnic groups.^[53]^ Finally, while we did not detect statistical heterogeneity, we cannot fully rule out that the selected SNPs might affect LTL through pathways other than serum copper levels (vertical pleiotropy), although the consistency of the effect across different MR methods provides some reassurance.^[54]^ Fourth, as noted, LTL is measured cross-sectionally, so our results do not directly inform about telomere attrition rates.

In conclusion, this MR study provides evidence for a positive causal effect of genetically predicted copper levels on LTL. These findings support the hypothesis that copper plays a role in cellular aging processes, potentially through antioxidant mechanisms.^[43,55]^ Future research should focus on experimental validation of the copper-superoxide dismutase-telomere pathway and investigate whether optimizing copper status can mitigate age-related telomere attrition in clinical settings.^[56]^

In conclusion, this MR study provides genetic evidence consistent with a potential positive causal effect of circulating copper levels on LTL. While initial univariable analyses suggested a possible link with vitamin B6, multivariable adjustment indicated that this was likely not an independent causal effect. No robust evidence was found to support causal associations for the other 13 micronutrients analyzed. These findings support a scientific hypothesis that copper may play a role in telomere maintenance, possibly through antioxidant pathways. However, these genetic associations should be interpreted with caution. Future mechanistic studies and well-powered randomized controlled trials are required to validate these findings and explore biological plausibility before drawing clinical inferences for human health.

## Acknowledgments

The authors thank all participants and investigators of the GWAS studies included.

## Author contributions

**Conceptualization:** Hehe Wang, Hanyu Zhu.

**Methodology:** Hanyu Zhu, Manman Chen.

**Software:** Hanyu Zhu, Hehe Wang.

**Supervision:** Hanyu Zhu.

**Investigation:** Manman Chen, Yaowen Wang.

**Resources:** Manman Chen, Chunlin Li, Hehe Wang.

**Project administration:** Yaowen Wang, Chunlin Li.

**Data curation:** Xiuqiang Zhang

**Formal analysis:** Xiuqiang Zhang, Chunlin Li.

**Visualization:** Hehe Wang.

**Writing – original draft:** Hanyu Zhu, Hehe Wang.

**Writing – review & editing:** Hanyu Zhu.




